# Relationship between Substantia Nigra Neuromelanin Imaging and Dual Alpha-Synuclein Labeling of Labial Minor in Salivary Glands in Isolated Rapid Eye Movement Sleep Behavior Disorder and Parkinson’s Disease

**DOI:** 10.3390/genes13101715

**Published:** 2022-09-24

**Authors:** Graziella Mangone, Marion Houot, Rahul Gaurav, Susana Boluda, Nadya Pyatigorskaya, Alizé Chalancon, Danielle Seilhean, Annick Prigent, Stéphane Lehéricy, Isabelle Arnulf, Jean-Christophe Corvol, Marie Vidailhet, Charles Duyckaerts, Bertrand Degos

**Affiliations:** 1 Centre National de la Recherche Scientifique CNRS, Institut National de la Santé Et de la Recherche Médicale INSERM, Institut du Cerveau ICM, Sorbonne Université, 47-83 Bd de l’Hôpital, 75013 Paris, France; 2 Clinical Investigation Center for Neurosciences, Department of Neurology, Assistance Publique Hôpitaux de Paris, Pitié-Salpêtrière Hospital, 47-83 Bd de l’Hôpital, 75013 Paris, France; 3 Centre of Excellence of Neurodegenerative Disease (CoEN), AP-HP, Pitié-Salpêtrière Hospital, 47-83 Bd de l’Hôpital, 75013 Paris, France; 4 Department of Neurology, Institute of Memory and Alzheimer’s Disease (IM2A), AP-HP, Pitié-Salpêtrière Hospital, 47-83 Bd de l’Hôpital, 75013 Paris, France; 5 Centre de NeuroImagerie de Recherche CENIR, Institut du Cerveau ICM, 47-83 Bd de l’Hôpital, 75013 Paris, France; 6 Institut du Cerveau-ICM, Team “Movement Investigations and Therapeutics” (MOV’IT), 47-83 Bd de l’Hôpital, 75013 Paris, France; 7 Department of Neuropathology, Assistance Publique Hôpitaux de Paris, Pitié-Salpêtrière Hospital, 47-83 Bd de l’Hôpital, 75013 Paris, France; 8 HYSTOMICS Platform, Centre National de la Recherche Scientifique-CNRS, Institut National de la Santé Et de la Recherche Médicale-INSERM, Institut du Cerveau-ICM, Sorbonne Université, 47-83 Bd de l’Hôpital, 75013 Paris, France; 9 Sleep Disorders Unit, Assistance Publique Hôpitaux de Paris, Pitié-Salpêtrière Hospital, 47-83 Bd de l’Hôpital, 75013 Paris, France; 10 Neurology Unit, Avicenne University Hospital, Hôpitaux Universitaires de Paris-Seine Saint Denis, Sorbonne Paris Nord, AP-HP, NS-PARK/FCRIN Network, 125 Route de Stalingrad, 93009 Bobigny, France; 11 Dynamics and Pathophysiology of Neuronal Networks Team, Center for Interdisciplinary Research in Biology, Collège de France, PSL University, 11 Place Marcelin Berthelot, 75005 Paris, France

**Keywords:** Parkinson’s disease, alpha-Synuclein, minor salivary gland biopsy, immunostaining, microscopy, neuromelanin

## Abstract

We investigated the presence of misfolded alpha-Synuclein (α-Syn) in minor salivary gland biopsies in relation to substantia nigra pars compacta (SNc) damage measured using magnetic resonance imaging in patients with isolated rapid eye movement sleep behavior disorder (iRBD) and Parkinson’s disease (PD) as compared to healthy controls. Sixty-one participants (27 PD, 16 iRBD, and 18 controls) underwent a minor salivary gland biopsy and were scanned using a 3 Tesla MRI. Deposits of α-Syn were found in 15 (55.6%) PD, 7 (43.8%) iRBD, and 7 (38.9%) controls using the anti-aggregated α-Syn clone 5G4 antibody and in 4 (14.8%) PD, 3 (18.8%) iRBD and no control using the purified mouse anti-α-Syn clone 42 antibody. The SNc damages obtained using neuromelanin-sensitive imaging did not differ between the participants with versus without α-Syn deposits (irrespective of the antibodies and the disease group). Our study indicated that the α-Syn detection in minor salivary gland biopsies lacks sensitivity and specificity and does not correlate with the SNc damage, suggesting that it cannot be used as a predictive or effective biomarker for PD.

## 1. Introduction

Parkinson’s disease (PD) is the second most common progressive neurodegenerative disorder after Alzheimer’s disease, affecting 1–2% of individuals over 60 years of age, and increasing to 4–5% of the population by the age of 85 years [1]. According to the clinical criteria of the Movement Disorders Society (MDS), the diagnosis of PD is based on typical motor symptoms such as bradykinesia, rigidity, and tremor. However, non-motor symptoms such as hyposmia, isolated rapid eye movement sleep behavior disorder (iRBD), oral and gastrointestinal dysfunction, and depression can precede the onset of motor symptoms by a decade. The accuracy of the clinical diagnosis of PD is estimated between 46% and 90% [2] and depends on prolonged clinical observation and clinical response to Levodopa. Therefore, there is a critical need for reliable diagnostic biomarkers.

The pathological hallmark of PD is the degeneration of dopaminergic neurons in the substantia nigra pars compacta (SNc) and the presence of intra-neuronal inclusions (Lewy bodies) enriched in misfolded alpha-Synuclein (α-Syn) [3]. The accumulation of α-Syn is not limited to the brain but also occurs in the peripheral autonomic nervous system that innervates the skin, olfactory mucosa, gastrointestinal tract, salivary glands, retina, adrenal gland, and heart in PD patients [3]. 

We investigated whether minor salivary gland biopsies (MSGBs) could be used as an early predictive biomarker for the diagnosis of PD by investigating the presence of misfolded α-Syn immunostaining in patients with early idiopathic PD and iRBD, a prodromal form of α-synucleinopathies (PD, dementia with Lewy bodies, and multiple systemic atrophy) compared to HC. In addition, we investigated whether the presence of α-Syn immunostaining in MSGB was associated with more severe motor or cognitive disorders or with the severity of lesions in the SNc using MRI measures. We explored the neurodegenerative changes in the SNc using neuromelanin-sensitive MRI in PD [4] and iRBD [5]. 

## 2. Materials and Methods

### 2.1. Standard Protocol Approval, Patient Consent, Funding

The study was sponsored by INSERM, approved by French regulatory authorities and by the local Committee on Ethics and Human Research, and conducted in accordance with the Declaration of Helsinki. All patients and HCs provided written informed consent (CPP Paris VI, RCB 2014-A00725-42) for inclusion. All participants were fully informed of assured anonymity, why the research was being conducted, how their data would be used, and the associated risk. The study was funded by a grant from the French State “Investissements d’Avenir” (ANR-10-IAIHU-06). Additional funding was obtained from a grant from the France Parkinson Association and EDF foundation.

### 2.2. Subjects

Participants were included in the ICEBERG study (ClinicalTrials.gov Identifier: NCT02305147), an ongoing observational, prospective, monocentric, four-year natural history study including patients with PD, iRBD, and HCs conducted at the Paris Brain Institute (Institut du Cerveau—ICM, Pitié-Salpêtrière Hospital, Paris, France). PD patients were defined according to International Movement Disorders Society (MDS) criteria and had less than 4 years disease duration at inclusion. All patients with iRBD were defined by sleep neurologists (I.A. and S.L.S.) using polysomnography (i.e., tonic muscle atonia greater than twice the minimal level during >50% of epochs, during >18% of REM sleep, and/or behaviors on video during REM sleep) following the criteria of the International Classification of Sleep Disorders, third edition (ICSD-3, 2014) [6]. All subjects are comprehensively assessed at baseline and every year thereafter. Subjects underwent clinical (motor, neuropsychiatric, sleep, ocular, and cognitive evaluations) and imaging assessments. In all participants, clinical, MRI, MSGB, and polysomnography evaluations were performed during the inclusion visit. All participants signed informed consent.

### 2.3. Clinical Assessment

The (part I–IV) Movement Disorders Society Unified PD Rating Scale (MDS-UPDRS) [7] was carried out in all groups by movement disorder specialists (M.V., G.M., and J.-C.C.). In the PD patients, the motor examination was carried out in the OFF-drug conditions, after a 12 h interruption of the antiparkinsonian medication, and in the ON-drug conditions after the administration of a single suprathreshold dose of antiparkinsonian medications. Dopaminergic drugs and doses were converted to levodopa-equivalent daily doses (LEDD) [8]. Cognition was assessed using the Montreal Cognitive Assessment (MoCA) (range 0–30) [9]. 

### 2.4. MRI Data Acquisition and Analysis

All subjects were scanned on a 3 Tesla Prisma MRI (Siemens, Erlangen, Germany) using a 64-channel head coil for signal reception. The MRI protocol included a whole-brain T_1_-weighted three-dimensional (3D) anatomical image acquired using magnetization-prepared two rapid gradient echoes (MP2RAGE) and a T_1_-weighted two-dimensional (2D) turbo spin echo (TSE) protocol for neuromelanin-sensitive imaging [4]. For the TSE acquisition, the transverse slices were oriented perpendicular to the long axis of the brainstem, and the field of view included both the SNc and the locus coeruleus. Briefly, two experienced raters blind to the subject’s clinical status manually segmented the SNc (S.L., R.G.). A background region comprising the tegmentum and superior cerebral peduncles was also manually traced in order to obtain the signal-to-noise ratio (SNR) by normalizing the mean signal in the SNc relative to the background signal as previously described [4]. A total of six scans were not analyzed either due to image artifacts (one PD patient) or the unavailability of MRI (three PD, one iRBD, and one HC). All analyses were performed using software programs written with an in-house algorithm in MATLAB 9.3 (version R2017b; MathWorks Inc, Natick, MA, USA), Statistical Parametric Mapping (SPM) (version SPM12, London, UK, http://www.fil.ion.ucl.ac.uk/spm, accessed on 20 September 2022), FreeSurfer (v5.3.0; MGH, Harvard, MA, USA, http://surfer.nmr.mgh.harvard.edu/, accessed on 20 September 2022), and FSL (Version 5.0; FMRIB Software Library, Oxford, UK).

### 2.5. Labial Salivary Gland Biopsy

After an anesthetic injection of lidocaine 2% into the inner side of the lower lip, a 0.5–1 cm horizontal incision was performed along the long axis of the lower lip mucosa just lateral to the midline while stretching the lip, allowing the removal of one–two accessory salivary glands with a scalpel, which were placed into a saline solution. Hemostasis was achieved by maintaining pressure with gauze. No antibiotics or pain medications were needed, and no adverse event was reported.

### 2.6. Immunohistochemistry

Small salivary gland biopsies were immersed in saline solution in a 4% buffered formalin solution for less than 72 h and embedded in paraffin. Serial 5 µm-thick sections were performed. To identify nerve fibers and α-Syn aggregates, immunohistochemistry was performed with the following antibodies: anti-phosphorylated high and medium molecular weight neurofilament (anti-NF) (clone SMI 310, mouse monoclonal, Biolegend, dilution 1/4000), anti-aggregated-α-Syn (clone 5G4, amino acids 46–53, mouse monoclonal, Merck, dilution 1/4000) [10], and anti-α-Syn (clone 42/Synuclein, amino acids 15–123, mouse monoclonal, BD Transduction Laboratories, dilution 1/4000) [11]. Immunohistochemistry was performed in a Nexes station automated system (Ventana Medical System, Inc., Roche, Basel, Switzerland). A pre-treatment was performed using a proprietary high pH buffer (pH8) (CC1). The detection of the anti-NF and the anti-α-Syn was performed with the UltraView Universal DAB Detection Kit (Ventana Medical System, Inc., Roche, Basel, Switzerland). The detection of the anti-aggregated-α-Syn was carried out with the OptiView DAB Immunohistochemistry Detection Kit (Ventana Medical System, Inc., Roche, Basel, Switzerland). The concordance between the two neuropathologists (C.D., S.B) who examined the slides blindly was 93%. The following variables were collected: lymphocytes infiltrate, α-Syn aggregates, and neurofilaments (Supplementary Figure S1). 

### 2.7. Statistical Analyses

In order to compare the SNc measurements and the presence of α-Syn deposits between PD, iRBD, and HCs, a general linear model (GLM) adjusted for age and gender was performed, with identity link and normal distribution for the former and with logit link and Bernoulli distribution for the latter. Post hoc Tukey HSD tests were performed when the group effect was significant. Using Wilcoxon–Mann–Whitney tests, we conducted separate analyses in the PD, iRBD, and HC groups to compare demographics, clinical score, and SNc measurements between subjects who showed α-Syn deposits either using α-Syn clone 5G4 or clone 42 antibodies to those who did not. Differences were considered significant at *p* < 0.05. Statistical analyses were performed using R 4.1.2. (R Foundation for Statistical Computing, Vienna, Austria. https://www.R-project.org/, accessed on 20 September 2022). 

## 3. Results

The study data were collected and managed using REDCap electronic data capture tools hosted at the ICM [12]. There was no age difference between the PD, iRBD, and HC groups, with a mean age of 64.5 ± 8.3 years old in the cohort (Table 1). There were more men in the iRBD group than in the PD and HC groups. The PD patients had a lower educational level than the iRBD patients. The PD patients had a mean age at disease onset of 61.5 ± 9 years old, disease duration of 21.7 ± 12.8 months, and took an LEDD of 342.3 ± 176.8 mg/d, including 77.4 ± 88.6 mg/d of dopamine agonists and 169.4 ± 178.7 mg/d of levodopa. Patients had iRBD for 88.3 ± 93.3 months and a mean age at iRBD onset of 59.6 ± 10.7 years old. The cognitive scores did not differ between the groups. The motor scores were worse in the PD than in the iRBD and HC groups. The non-motor symptom scores of PD and iRBD patients differed from those of HC and were similar between PD and iRBD for the Scales for Outcomes in Parkinson’s disease—Autonomic (SCOPA-AUT), Non-Motor Symptoms Scale (NMSS), and the University of Pennsylvania Odor Identification Test (UPSIT)—and differed from the HC group as reported in Table 1. Deposits of α-Syn were detected in nerve fibers of the minor salivary glands in 15 (55.6%) PD patients, (43.8%) iRBD patients, and 7 (38.9%) HCs using the anti-aggregated α-Syn clone 5G4 antibody and in 4 (14.8%) PD patients, 3 (18.8%) iRBD patients, and no HC using the purified mouse anti-α-Syn, clone 42 antibody (Supplementary Figure S1). No difference was found between the three groups (α-Syn clone 5G4 antibody, *p* = 0.49; anti-α-Syn, clone 42 antibody *p* = 0.12). The SNRs were lower in the PD than in the other groups (mean difference estimate (MDE) ± standard error (SE), vs. controls: −1.90 ± 0.61, post hoc *p* = 0.009; vs. iRBD patients: −1.73 ± 0.68, post hoc *p* = 0.037) (Table 1). The volumes of the SNc were also lower in PD and iRBD than in the controls but the difference did not reach significance (Supplementary Figure S2). The comparison between subjects with α-Syn deposits and subjects without α-Syn deposits in each group did not differ for disease course, as well as clinical motor and non-motor symptoms scores, except for the olfaction scores, which were higher in PD patients with misfolded α-Syn deposits (Table 2). In addition, no difference in SNc volume and SNR were found in PD and iRBD, suggesting that there was no relationship between neuromelanin loss and α-Syn deposition as evaluated by MSGB (Table 2). 

## 4. Discussion

In this study, misfolded α-Syn deposits were found in the salivary glands of two-thirds of the PD patients, half of the patients with iRBD, but surprisingly also in more than one-third of HC, which is higher than in most previous studies [13,14], indicating that these measures lack specificity at this age. These cases may be considered “incidental or preclinical cases” given that post-mortem studies in the brain have shown Lewy body pathology in about 10–20% of people over the age of 60 without parkinsonism or dementia during their lifetime [15].

There were no differences for any SNc measures between subjects with vs. without α-Syn deposits, regardless of the antibody used for the clinical group. The volumes of the SNc were also lower in PD and iRBD than in HCs but the difference did not reach significance. These results indicate an absence of a link between neuromelanin loss in the SNc and α-Syn deposits in the salivary glands. 

Although the procedure was safe, our results suggested that MSGB lacks sufficient accuracy to detect α-Syn deposits in salivary glands in PD and in iRBD, thus it cannot be considered a useful and relevant biomarker of synucleinopathy. The sensitivity and specificity of the measures of α-Syn deposits in the MSGB were disappointing, as reported in unlike previous studies. 

The abnormal accumulation of α-Syn around gland cells was reported in minor salivary glands in PD patients but in none of the HCs [16,17]. Another study found an abnormal accumulation of α-Syn in 3 out of 16 PD patients, and 2 out of 11 HCs exhibited weak phosph-α-Syn [13]. In another study, the ratio of nerve fibers immunoreactive to α-Syn was slightly decreased in seven PD patients as compared to seven HCs [18]. Conversely, Ser129-phosphorylated-α-Synuclein immunoreactive nerve fibers were identified in five of seven PD cases but no HCs [18]. Phosph-α-Syn was detected in 31 of 62 patients with iRBD, in 7 of 13 patients with PD, in 5 of 10 patients with dementia with Lewy bodies, and in 1 of the 33 HCs [14]. 

The differences between our results and those of previous studies using MSGB are likely due to different methods used to search peripheral Lewy bodies, including biological specimen collection techniques, biopsy locations, histological methods, observation, and experience criteria. 

Many studies have shown that submandibular gland biopsy is more sensitive and specific than the MSGB in the diagnosis of PD. α-Syn aggregates were detected in the nerve fibers of the glandular parenchyma in 8 (89%) of 9 patients with iRBD and 8 (67%) of 12 with PD, but none of the HCs [19]. In an autopsy-based study of submandibular glands, Lewy pathology was present in all PD patients (nine of nine cases) and incidental Lewy body disease (two of three cases) but not in multiple system atrophy or HC [11]. Using sections of large segments (simulating open biopsy) and needle cores of submandibular glands from 128 subjects, immunoreactive phosph-α-Syn-positive nerve fibers were present in all 28 PD patients and three patients with Alzheimer’s disease and Lewy bodies, but none in HCs. Cores from frozen submandibular glands were positive for phosph-α-Syn in 17 of 19 PD patients [20]. 

Based on these results, needle core biopsy of submandibular glands and MSGB was performed in patients with PD showing 9/12 biopsies positive for phosph-α-Syn (75%) while only 1/15 MSGB were positive (6.7%) [21]. Subsequently, a study of submandibular gland needle biopsies from patients with early PD (<5 years of disease duration) showed positive staining in 14 of 19 patients (74%) and only 2 of 9 HCs (22%) [22].

Another factor of variability comes from the wide range of available antibodies that have been used in the different studies depending on the divergent fixation, epitope exposure, and signal development methods [23]. It is noteworthy that some studies looked for α-Syn and others for phosph-α-Syn. However, it became clear that peripheral α-Syn is detectable even in HCs. Therefore, previous studies suggested using antibodies against phosph-α-Syn with proteinase K pretreatment, since phosph-α-Syn is expressed at very low levels in normal controls, and proteinase K is able to digest normal α-Syn. They concluded that phosph-α-Syn is the best hallmark for α-Synucleinopathy [20]. 

Baseline reductions in neuromelanin-based SN volume and signal intensity were observed in early PD (although not significant), as reported previously in de novo patients [24] and early PD [25] and in line with histological studies [26]. In previous studies, volume reductions correlated positively with disease duration [27,28]. iRBD patients also displayed lower neuromelanin volume and signal intensity than healthy controls [4,5]. The lack of significance in PD and iRBD was probably due to the small number of subjects [4]. 

## 5. Limitation of Study

Our study has several limitations including a small sample size of each subgroup and a small number of labial salivary glands analyzed. The short disease duration of PD and the realization of the MSGBs at the time of the inclusion visit are limiting factors in interpreting our results. It would have been interesting to perform again MSGBs at a later stage to investigate whether the presence of peripheral α-Syn changed over time. The choice of antibodies could also explain the differences observed between studies. For our study, we performed immunohistochemistry with two -Syn antibodies: (a) clone 42/-synuclein [11], which is a highly sensitive antibody and has been previously described to detect -Syn pathological aggregates in the submaxillary gland and peripheral nervous system, (b) 5G4 clone [10] which also has high sensitivity and detects the oligomeric and fibrillary forms of -Syn but not the normal soluble monomeric form of the protein. The latter was employed in substitution of the phosph-α-Syn, expecting to observe a higher number of cases with pathology while preserving the specificity of the detection of the pathological aggregates.

## 6. Conclusions

In recent years, a major research effort has been made to find biomarkers that would allow an accurate and early diagnosis of synucleinopathies. Recent immunohistochemistry studies have demonstrated, with varying success, that accumulation of α-Syn also occurred in the peripheral autonomic nervous system that innervates the skin, olfactory mucosa, gastrointestinal tract, retina, adrenal gland, and heart in PD [3] as well as in human fluid (saliva, red blood cells, and cerebrospinal fluid) in iRBD patients [29,30]. Therefore, further explorations aimed at studying the pathological protein by non-immuno-histochemical techniques and characterizing the inflammatory infiltrate in peripheral tissues from multiple organs in combination with human fluid are needed. Novel methods for pathological α-Syn detection in human tissues, including real-time quaking-induced conversion (RT-QuIC) and protein misfolding cyclic amplification (PMCA), seem to have higher diagnostic sensitivity.

## Figures and Tables

**Table 1:** Demographic, clinical, biopsies, and MRI measures in patients with Parkinson’s disease (PD) and with isolated rapid eye movement sleep disorder (iRBD) as well as in healthy controls.

	PD (a)	iRBD (b)	Healthy Controls (c)	*p* ‡
Number	27	16	18	0.003 *
**Demographics**				
Gender (Male)	17 (63%) **^b^**	15 (94%) **^a,c^**	7 (39%) **^b^**	0.003 *
Age (years)	63.3 ± 8.9	68.1 ± 5.7	62.93 ± 9.0	0.124
Educational level	5.7 ± 1.6 **^b^**	6.8 ± 0.5 **^a^**	6.6 ± 0.8	0.024 *
**Clinical data**				
Age at onset (years)	61.5 ± 9.0	59.6 ± 10.7	NA	NA
Disease duration (months)	21.7 ± 12.8	88.3 ± 93.3	NA	NA
Dopaminergic agonists (mg)	77.4 ± 88.6	NA	NA	NA
Levodopa (mg)	169.4 ± 178.7	NA	NA	NA
LEDD (mg)	342.3 ± 176.8	NA	NA	NA
**Clinical scores**				
Montreal Cognitive Assessment ∫	27.6 ± 2.1	27.8 ± 2.3	27.8 ± 1.5	0.986
MDS-UPDRS I	9.7 ± 3. 7 **^c^**	7.50 ± 3.1 **^c^**	5.06 ± 2.7 **^a,b^**	<0.001 *
MDS-UPDRS II	8.5 ± 3.4 **^b,c^**	1.4 ± 1.1 **^a^**	0.8 ± 1.3 **^a^**	<0.001 *
MDS-UPDRS III Off Med	31.6 ± 9.2 **^b,c^**	9.9 ± 4.1 **^a^**	6.1 ± 7.1 **^a^**	<0.001 *
MDS-UPDRS III On Med	27.6 ± 7.3	NA	NA	NA
MDS-UPDRS IV	0.2 ± 0.8	0.00 ± 0.00	0.00 ± 0.00	0.365
Hoehn and Yahr	2.0 ± 0.2 **^b,c^**	0.4 ± 0.8 **^a^**	0.2 ± 0.7 **^a^**	<0.001 *
SCOPA-AUT	11.7 ± 6.3 **^c^**	10.0 ± 5.6	5.6 ± 4.2 **^a^**	0.002 *
NMSS scale	8.0 ± 3.9 **^c^**	8.9 ± 3.7 **^c^**	3.2 ± 3.1 **^a,b^**	<0.001 *
UPSIT	22.0 ± 5.3 **^c^**	19.6 ± 5.0 **^c^**	32.6 ± 6.4 **^a,b^**	<0.001 *
**α-Syn deposits, No with (%)**				
α-Syn clone 5G4 antibodies	15 (55.6%)	7 (43.8%)	7 (38.9%)	0.491
α-Syn clone 42 antibodies	4 (14.8%)	3 (18.8%)	0 (0.00%)	0.115
either α-Syn clone 5G4 or 42 antibodies	17 (63.0%)	8 (50.0%)	7 (38.9%)	0.286
**SNc measurements**				
Volume (mm^3^)	234.4 ± 45.4	244.8 ± 18.4	257.7 ± 56.1	0.228
Signal-to-Noise Ratio (SNR)	109.8 ± 1.5 **^b,c^**	111.6 ± 2.1 **^a^**	111.7 ± 2.1 **^a^**	0.004 *

Data are given as mean ± standard deviation for continuous variables and as numbers (percentages) for categorical variables. * The level of significance was set at *p* < 0.05. ‡ For demographics, the Kruskal–Wallis test was used to compare the groups for continuous variables and Fisher’s exact test for categorical variables. Post hoc comparisons were performed using pairwise Wilcoxon–Mann–Whitney tests for continuous variables and pairwise Fisher’s exact tests for qualitative variables. Benjamini Hochberg correction was applied to correct for multiple testing. For clinical scores and SNc measurements, linear models adjusted for age and sex were performed. For α-Syn deposits, logistic regressions adjusted for age and sex were performed. Post hoc Tukey HSD tests were performed for pairwise comparison. ∫ Linear model with MoCA as the dependent variable was adjusted for age, sex, and educational level. ^a^ differs from the PD group; ^b^ differs from the iRBD group; ^c^ differs from controls. Abbreviations: LEDD Levodopa Equivalent Daily Dose; MoCA = Montreal Cognitive Assessment; MDS-UPDRS = Movement Disorders Society Unified Parkinson’s Disease Rating Scale; SCOPA-AUT = Scales for Outcomes in Parkinson’s disease—Autonomic; NMSS = Non-Motor Symptoms Scale; UPSIT University of Pennsylvania Smell Identification Test. Sociocultural level score ranges from 0–7. Cognition score ranges: MoCA total score 0–30; (high scores = better cognitive performances). Motor score ranges: MDS-UPDRS score ranges: MDS-UPDRS I 0–52, MDS-UPDRS II 0–52, MDS-UPDRS III 0–132, MDS-UPDRS IV 0–24 (high scores = worse clinical assessment); Hoehn and Yahr staging 0–5 (low scores = better clinical assessment); Non-motor score ranges: SCOPA-AUT 0–69; NMSS 0–30 (high scores = worse autonomic functions); UPSIT 0–40 (low scores = worse smell assessment).

**Table 2:** Comparison between subjects with α-Syn deposits and subjects without α-Syn deposits in each clinical group.

	PD			iRBD			Healthy Controls		
	With α-Syn Deposits	Without α-Syn Deposits	*p* ‡	With α-Syn Deposits	Without α-Syn Deposits	*p* ‡	With α-Syn Deposits	Without α-Syn Deposits	*p* ‡
Demography Number (%)	17 (63%)	10 (37%)		8 (50%)	8 (50%)		7(39%)	11 (61.1%)	
Gender (Male)	10 (58.8%)	7 (70.0%)	0.69	8 (100.0%)	7 (87.5%)	1.000	3 (42.9%)	4 (36.4%)	1.000
Age (years)	64. ± 9.4	61.8 ± 8.4	0.547	69.1 ± 4.8	67.2 ± 6.6	0.600	59.8 ± 10.3	64.9 ± 7.9	0.342
Educational level	5.6 ± 1.8	5.9 ± 1.4	0.643	6.7 ± 0.7	6.9 ± 0.3	0.927	6.71 ± 0.49	6.5 ± 0.9	0.954
**Clinical data**									
Age at onset (years)	62.5 ± 9.4	59.7 ± 8.5	0.482	59.8 ± 13.0	59.4 ± 8.8	0.565	NA	NA	NA
Disease duration (months)	19.7 ± 12.6	24.9 ± 13.2	0.248	100.0 ± 118.8			NA	NA	NA
Dopaminergic agonists	50.2 ± 88.7	118.1 ± 74.8	0.019 *	NA	NA	NA	NA	NA	NA
Levodopa	139.7 ± 180.1	219.9 ± 173.5	0.237	NA	NA	NA	NA	NA	NA
LEDD	291.8 ± 175.3	418.0 ± 157.8	0.211	NA	NA	NA	NA	NA	NA
**Clinical scores**									
MoCA (/30)	27.6 ± 2.2	27.5 ± 1.9	0.738	28.2 ± 1.6	27.2 ± 3.0	0.591	27.6 ± 1.7	28.0 ± 1.4	0.577
MDS-UPDRS I	9.6 ± 4.2	9.9 ± 2.7	0.631	6.2 ± 3.3	8.7 ± 2.4	0.064	5.6 ± 3.1	4.7 ± 2.4	0.291
MDS-UPDRS II	8.3 ± 3.6	8.8 ± 3.3	0.577	1.9 ± 1.2	0.9 ± 0.8	0.091	0.6 ± 1.0	0.9 ± 1.4	0.665
MDS-UPDRS III Off Med	30.8 ± 9.9	32.9 ± 8.2	0.258	8.0 ± 4.2	11.7 ± 3.1	0.071	2.57 ± 2.1	8.3 ± 8.4	0.102
MDS-UPDRS III On Med	27.2 ± 7.1	28.2 ± 8.0	0.731						
MDS-UPDRS IV	0.3± 1.0	0.0 ± 0.0	0.269	0.0 ± 0.0	0.0 ± 0.0		0.0 ± 0.0	0.0 ± 0.0	
Hoehn and Yahr	2.1 ± 0.2	2.0 ± 0.0	0.443	0.6 ± 0.9	0.2 ± 0.7	0.298	0.0 ± 0.0	0.3 ± 0.9	0.425
SCOPA-AUT	13.1 ± 7.1	9.4 ± 3.9	0.158	10.4 ± 5.4	9.9 ± 5.9	0.791	6.3 ± 5.4	5.2 ± 3.5	0.964
NMSS	8.1 ± 4.0	7.8 ± 3.8	0.899	8.1 ± 3.5	9.7 ± 4.0	0.337	3.3 ± 3.4	3.2 ± 3.0	0.927
UPSIT	24.3 ± 4.3	18.2 ± 4.7	0.003 *	19.1 ± 2.8	20.1 ± 6.7	0.833	34.0 ± 3.5	31.7 ± 7.8	0.728
**SNc measurements**									
Volume (mm^3^)	242.3 ± 47.6	224.0 ± 42.5	0.535	247.1 ± 19.54	242.99 ± 18.64	0.699	255.4 ± 53.9	259.6 ± 61.01	0.958
Signal-to-Noise Ratio (SNR)	109.9 ± 1.6	109.7 ± 1.5	0.770	112.2 ± 2.0	110.9 ± 2.1	0.418	111.4 ± 1.3	111.9 ± 2.6	0.380

Notes. Data are given as mean ± standard deviation for continuous variables and as count (percentages) for categorical variables. * The level of significance was set at *p* < 0.05. ‡ Wilcoxon–Mann–Whitney tests were used to compare groups for continuous variables and Fisher’s exact test for categorical variables. Abbreviations: LEDD Levodopa Equivalent Daily Dose; MoCA = Montreal Cognitive Assessment; MDS-UPDRS = Movement Disorders Society Unified Parkinson’s Disease Rating Scale; SCOPA-AUT = Scales for Outcomes in Parkinson’s disease—Autonomic; NMSS = Non-Motor Symptoms Scale; UPSIT = University of Pennsylvania Smell Identification Test. Sociocultural level score ranges from 0–7. Cognition score ranges: MoCA total score 0–30; (high scores = better cognitive performances). Motor score ranges: MDS-UPDRS score ranges: MDS-UPDRS I 0–52, MDS-UPDRS II 0–52, MDS-UPDRS III 0–132, MDS-UPDRS IV 0–24 (high scores = worse clinical assessment); Hoehn and Yahr staging 0–5 (low scores = better clinical assessment); Non-motor score ranges: SCOPA-AUT 0–69; NMSS 0–30 (high scores = worse autonomic functions); UPSIT 0–40 (low scores = worse smell assessment).

## Data Availability

Due to its proprietary nature, supporting data cannot be made openly available. For further information about the data and conditions for access, you can contact by email the Principal Investigator of ICEBERG study (Pr Marie Vidailhet: marie.vidailhet@aphp.fr).

## Supplementary Materials

The following supporting information can be downloaded at: https://www.mdpi.com/article/10.3390/genes13101715/s1, Figure S1: Immunohistochemistry of minor salivary glands in a PD patient. Figure S2: Manual tracing of the SNc in neuromelanin-sensitive images: neuromelanin images of a representative PD (left column), an iRBD patient (middle column) and an HC (right column).
