# Supplementary material for: Relationship between Substantia Nigra Neuromelanin Imaging and Dual Alpha-Synuclein Labeling of Labial Minor in Salivary Glands in Isolated Rapid Eye Movement Sleep Behavior Disorder and Parkinson’s Disease
Source: Genes (Basel). 2022 Sep 24;13(10):1715. doi: 10.3390/genes13101715 (PMC9601642; doi:10.3390/genes13101715)

SUPPLEMENTARY MATERIALS

Supplementary Figure S1: Immunohistochemistry of minor salivary glands in a PD patient. Magnification of a minor salivary gland and its detailed histological structure stained with hematoxylin-eosin characterized by the presence of dense aggregates with 50 or more lymphocytes, usually located in the perivascular area, in 4 mm^2 of glandular tissue according to Chisholm-Mason's score **(a)**. Immunohistochemistry with anti-neurofilament clone SMI 310: small nerves with diffuse immunoreactivity along axons, brown stain in **(b)**, and with anti-α-Synuclein antibody, purified mouse anti-α-Syn clone 42: a Lewy body deposit, black arrow in **(c)**.

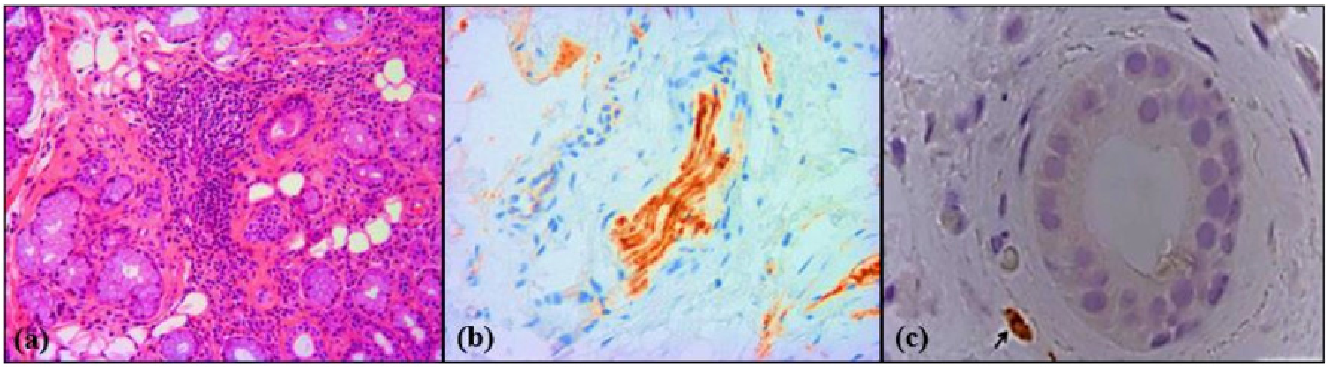

Supplementary Figure S2: Manual tracing of the SNc in neuromelanin-sensitive images: neuromelanin images of a representative PD (left column), an iRBD patient (middle column) and an HC (right column). The first row shows images with the manual tracing of the left and right SNc regions of interest (ROI) (left SNc in red and right SNc in green) superimposed on the neuromelanin-sensitive image and second row shows images without ROI. All SNc measurements significantly differed between HC, PD and iRBD patients at inclusion. The SNR were lower in the PD than in other groups (mean difference estimate (MDE) ± standard error (SE), vs. HC: -1.90±0.61, post-hoc p=0.009; vs. iRBD patients: -1.73±0.68, post-hoc p=0.037). Volumes of the SNc were also lower in PD and iRBD than in HC but the difference did not reach significance (p=0.228).

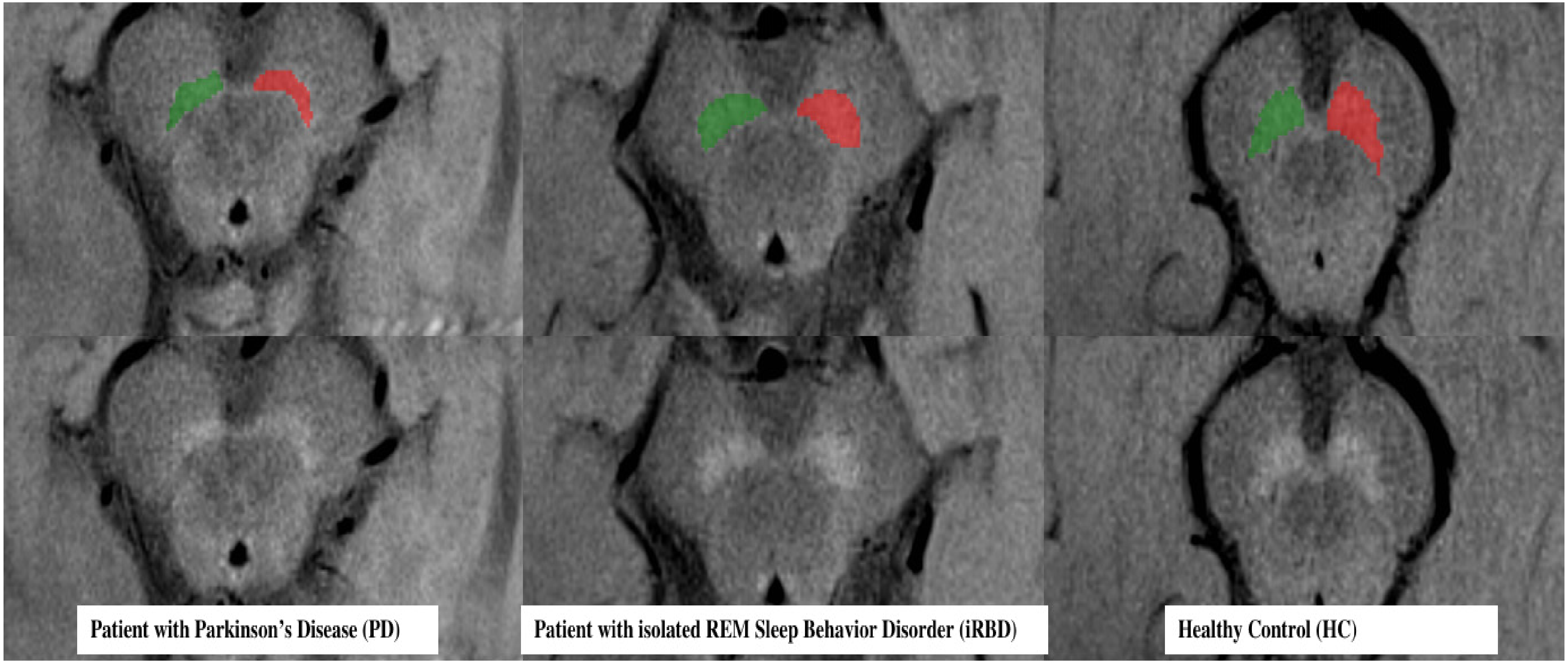

Supplement: Supplementary file 1 [file genes-13-01715-s001.zip › genes-1861425-supplementary.pdf]
